# In the Age of Viral Pandemic, Can Ingredients Inspired by Human Milk and Infant Nutrition Be Repurposed to Support the Immune System?

**DOI:** 10.3390/nu13030870

**Published:** 2021-03-06

**Authors:** Lauren R. Brink, Maciej Chichlowski, Nitida Pastor, Athmaram Thimmasandra Narayanappa, Neil Shah

**Affiliations:** 1 Medical and Scientific Affairs, Nutrition, Reckitt Benckiser, Evansville, IN 47721, USA; maciej.chichlowski@rb.com (M.C.); nitida.pastor@rb.com (N.P.); 2 Microbiome Management Science Platform, Reckitt Benckiser, Montvale, NJ 07645, USA; Athmaram.ThimmasandraNarayanappa@rb.com; 3 Medical and Scientific Affairs, Nutrition, Reckitt Benckiser, Slough SL1 3UH, UK; neil.shah@rb.com; 4 University College London, Great Ormond Street, London WC1N 3JH, UK

**Keywords:** anti-viral, infant nutrition, lactoferrin, milk fat globule membrane, osteopontin, glycerol monolaurate, human milk oligosaccharides, probiotics, postbiotics, polyunsaturated fatty acids

## Abstract

In 2020, with the advent of a pandemic touching all aspects of global life, there is a renewed interest in nutrition solutions to support the immune system. Infants are vulnerable to infection and breastfeeding has been demonstrated to provide protection. As such, human milk is a great model for sources of functional nutrition ingredients, which may play direct roles in protection against viral diseases. This review aims to summarize the literature around human milk (lactoferrin, milk fat globule membrane, osteopontin, glycerol monolaurate and human milk oligosaccharides) and infant nutrition (polyunsaturated fatty acids, probiotics and postbiotics) inspired ingredients for support against viral infections and the immune system more broadly. We believe that the application of these ingredients can span across all life stages and thus apply to both pediatric and adult nutrition. We highlight the opportunities for further research in this field to help provide tangible nutrition solutions to support one’s immune system and fight against infections.

## 1. Nutrition and the Immune System

It is well recognized that adequate nutrition is essential for robust immune responses. In clinical settings, ensuring patients remain nourished is essential to obtaining disease resolution and supporting critically ill patients [1,2,3]. The role of vitamins and minerals for an adequately functioning immune system has also been thoroughly examined. Many nutrients, including vitamin D, antioxidant vitamins such as A and C, and minerals such as zinc have demonstrated roles in maintaining a healthy immune system [4,5,6,7,8]. Human milk (HM) contains both nutritive and non-nutritive components and is the gold standard for understanding infant nutrition. The World Health Organization (WHO) recommends exclusive breastfeeding for the first 6 months of life, with continued breastfeeding up to two years of age or longer [9]. This guidance has not changed in relation to risk of severe acute respiratory syndrome coronavirus 2 (SARS-CoV-2) infection; while the possibility of vertical transmission has been shown to be minimal, it remains an active area of ongoing research [10]. It is important to point out that per recent guidelines from the Center for Disease Control (CDC), breast milk is not a likely source of SARS-CoV-2 transmission [11]. In addition, SARS-CoV-2 experimentally added to breast milk is inactivated via pasteurization [12]. Furthermore, breastfeeding has been associated with lowered incidence of all-cause and infection-related mortality in infants [13], and multiple components of HM have been investigated for immunologic benefit. A recent review on HM described microbiome, immunologic and metabolic factors as all having a role in attenuating early life intestinal inflammation [14]. Fatty acids present in HM have also recently been described to have protective effects [15]. Several HM components can now be isolated from dairy and recombinant sources, primarily with the aim of incorporation into infant formula to better approximate optimal HM. However, these ingredients may also have benefits across the entire lifespan. Thus, we aim to investigate the effect of ingredients inspired by HM to support the immune system (or natural defense) to reduce the risk of viral infection.

Viruses are the most common cause of acute respiratory and gastrointestinal (GI) diseases and influencing the immune system through nutrition could have enormous consequences for combating viral infections in infants and children. Influenza viruses and respiratory syncytial virus (RSV) are major causative agents of both upper and lower respiratory tract infections (RTIs) [16,17]. Rotaviruses are a leading cause of severe dehydrating gastroenteritis in children under the age of 5 years [18]. Further, neonatal rotavirus infections have been associated with severe GI diseases, including diarrhea, feed intolerance, and necrotizing enterocolitis (NEC) [19]. Noroviruses are small, non-enveloped viruses of the Caliciviridae family. They are single-stranded RNA viruses that cause most cases of acute gastroenteritis in all age groups [20]. Rotavirus and norovirus are examples of non-enveloped or “naked” viruses, in which glycoproteins facilitate entry of the virus into a new cell by recognizing and binding host cell receptors [21]. Such non-enveloped viruses are distinct from enveloped viruses, which include the outer lipid layer obtained from the host cell via the lysis or lysogenic process. Influenza virus is an example of an enveloped and single-stranded RNA virus belonging to the Orthomyxoviridae family. Recently, several new viruses associated with respiratory diseases have emerged, such as human bocavirus, human metapneumovirus, and the new coronaviruses [22]. The novel SARS-CoV-2 is now a global pandemic with vaccine and drug therapies currently in development. COVID-19 is the infectious disease caused by SARS-CoV-2, unknown before the outbreak in December 2019 originating in Wuhan, China and believed to derive from a bat virus [23]. Individuals at highest risk of serious consequences of SARS-CoV-2 infection include those with pre-existing conditions and the elderly [24]. SARS-CoV-2 infection can remain asymptomatic in up to 60% of individuals [25], meaning that the number of infected individuals is expected to be much higher than official reports.

Although classified as a respiratory virus, SARS-CoV-2 infection appears to also have implications within the GI tract. For instance, SARS-CoV-2 infects cells by binding its proteins to the angiotensin-converting enzyme 2 receptor (ACE2) [26]. This receptor is expressed in both the GI tract and lungs and plays a role in the acute inflammatory process triggered by SARS-CoV-2; characterized by mucosal infiltration of macrophages, neutrophils, and T-cells [27]. SARS-CoV-2 was detected in the stool of COVID-19 patients [28], suggesting that the GI tract can be a reservoir for viral replication and infection. Indeed, GI symptoms were the predominant presenting complaint in 20% of COVID-19 patients studied in a multi-center trial within the United States [29]. GI upset may be an early sign of infection, as a recent report demonstrated that internet searches on GI symptoms predicted a rise in COVID-19 cases weeks later [30].

**Figure 1:**
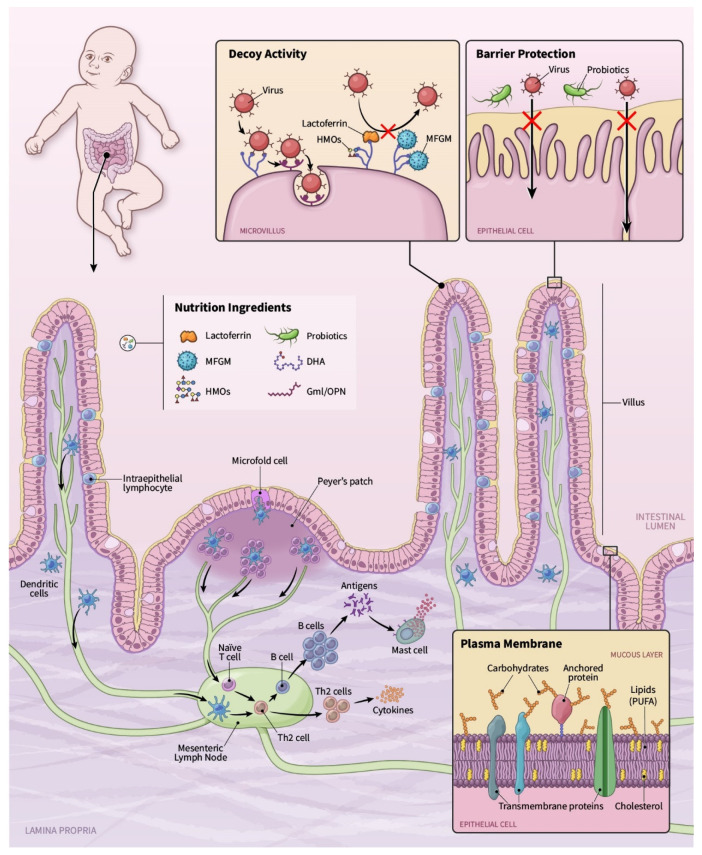
Legend: Functional nutrition ingredients can provide anti-viral activities when consumed and made available within the GI tract. Anti-viral activities can occur through reducing infectivity and binding to the gut epithelial cells. Ingredients can provide decoy activity such that they provide viral receptor binding limiting the ability of virus’ cell adhesion and infection. Ingredients can also support anti-viral infectivity through stimulating the local (and systemic) immune system response. Ingredients interact with intestinal immune cells through dendritic cell or M cell sampling. This sampling goes on to influence T and B cell production within the mesenteric lymph nodes, leading to a modified adaptive immune response, as measured through altered T/B cells, secretory IgA as well as inflammatory cytokines. Ingredients may also stimulate the gap junction protein function leading to decreased translocation of bacterial and viral products. The plasma membrane is the integral interface from which virus’ interact with cells. Modifications to the gut-associated lymphoid tissue inform the broader systemic immune system response. Thus, modulating the immune system through nutrition may be an effective way to provide broad anti-viral support.

Due to this global infectious challenge, there is a heightened interest in how nutrition can play a role in both the prevention of and susceptibility to infection. Potential nutritional interventions for COVID-19 were recently reviewed and include: Vitamin A, C, D, E and B vitamins, zinc, selenium, iron and omega-3 polyunsaturated fatty acids (PUFAs) [31]. In this review, we will focus on ingredients beyond vitamins and minerals, which are inspired by HM research and early life nutrition. Figure 1 provides a graphical overview of the concepts presented in this review. We specifically focus on functional, HM and infant nutrition-based ingredients for their ability to support the immune system and potentially reduce the risk and impact of viral infections, including SARS-CoV-2. In addition, these dietary interventions may also benefit the immune system through the promotion of an optimal gut microbiota and the improvement of vaccine efficacy.

## 2. Ingredients Inspired by Human Milk

### 2.1. Lactoferrin

Lactoferrin (Lf) is an iron binding protein within HM that has been demonstrated to be effective in supporting resistance to bacterial and viral infections as well as modulating the immune system [32,33]. Lf is also naturally found in mucosal secretions and is secreted by neutrophils during an active infection [34]. It can also be taken as a supplement, where it then acts as a nutraceutical or functional food to support host immunity against bacterial and viral infections. Lactoferrin can be isolated from dairy milk as well as produced as a recombinant protein. It is currently added to some infant formulas across the globe [35] in addition to being utilized in other oral supplementation and skin care products. Supplemental Lf has been examined for its role in tumorigenic processes [36], improving bone [37] and skin health (particularly wound healing [38], acne, psoriasis and diabetic ulcers [39]), as a nutritional solution/intervention for iron deficiency anemia [40] and for its immune-supporting properties, including anti-viral, bacterial, fungal and yeast [41,42,43,44]. Clinical evidence shows an effect of Lf on the health of infants through adulthood. A review published in 2012 identified 19 nutrition intervention clinical studies utilizing either human or bovine Lf in children [45]. For adults, effects on viral infections and general immune modulation have been studied [32].

#### 2.1.1. Viruses Studied and Proposed Mechanisms

Lf has strong anti-viral activity against a broad spectrum of non-enveloped and enveloped DNA and RNA viruses [46]. In vitro and in vivo anti-viral activity of Lf has been demonstrated against several viruses, including Herpes Simplex Virus (HSV) [47], Feline Herpes Virus (FHV) [48,49], Encephalomyocarditis virus [50], rotavirus [51,52], Mayaro virus [53], Coronavirus [54], cytomegalovirus [55], Influenza virus [52,56], Echovirus [57,58,59], Human Immunodeficiency virus (HIV) [60], Hepatitis B virus (HBV) and Hepatitis C virus (HCV) [61,62], Dengue virus [63], Poliovirus [64], Human papillomavirus (HPV) [65], Chikungunya virus (CHIKV) and Zika virus [66] Shielding virus-host interactions by directly binding to viral particle and inhibition of viral replication in host cells through immune cell/cytokine activation is the primary mechanism through which Lf exerts anti-viral activity. Lf inhibits the entry of viral particles into host cells, either by direct attachment to the viral particles or by blocking their cellular receptors [46].

Lf is proposed to exert its main biological activity following interaction with their receptors generally referred to as “lactoferrin receptor” on the target cells detected in multiple tissues and cell types including intestinal epithelial cells and lymphocytes [67,68]. There are several known Lf receptors viz., LDL receptor-related protein-1 (LRP-1) [69,70,71], Toll-like receptor-2 (TLR-2), TLR-4 and cytokine receptor 4 (CXCR4) [72], CD14 [68], intelectin-1 [73], Lf is also known to bind to heparan sulfate proteoglycans (HSPGs) that are present on cell-surface and extracellular matrix macromolecules made up of core protein decorated with covalently linked glycosaminoglycan (GAG) chains [74,75,76]. The widespread effects of Lf are attributed to its multiple receptors with multiple targets simultaneously hit, resulting in major effects [77,78].

The entry of bacteria, bacterial products or viruses into host cells evokes signaling pathways that involve mitogen-activated protein kinase (MAPK) [79], NF-κB [80], activator protein 1 (AP-1) [81], and various interferon regulatory factors (IRFs) [82]. Lf helps in activating the host defense mechanisms by binding to these receptors and also HSPG on cells during bacterial and viral infections, which leads to the activation of a complex biomolecular network through phosphorylation of relevant substrates (e.g., Transcription factors, histones, enzymes, microtubules) [83]. It is also proposed that viruses [84,85], as well as bacteria [86], binds to HSPGs, using this proteoglycan as entry into the cell. As previously demonstrated, HIV-tat protein, released from virus infected cells enters surrounding cells using HSPGs [74,76]. Lf is known to compete with tat proteins for receptor occupancy [87,88], and therefore plays a vital role in host immunity against HIV [89]. In COVID-19 infection, Lf may have a role to play in not only sequestering iron and inflammatory molecules that are severely increased during the cytokine storm, but also possibly by assisting in occupying receptors and HSPGs to prevent virus binding.

#### 2.1.2. Preclinical Evidence

Efficacy of Lf against both enveloped and non-enveloped viruses has been examined (as reviewed [41,46,90,91]). Direct binding to viral envelope proteins by Lf has been demonstrated for multiple viruses. Virus-dependent binding has been ascribed to Lf lobe terminals (N- and C-) and reliant on charge interactions. In HCV, binding was associated with the N-lobe terminus of lactoferrin [46]. The Lf N-lobe has also been described for its ability to bind to glycosaminoglycans, including heparan sulphate and chondroitin sulphate, which are common binding receptors for enveloped proteins. A dose dependent effect of Lf and SARS-Cov-1 infectivity was recently demonstrated. In this study, SARS pseudovirus was incubated with HEK293E/ACE2-Myc (human embryonic kidney) cells. The ability for Lf to inhibit infectivity of SARS-CoV-1 was removed when the virus particles were first incubated with heparin sulfate, which removed the heparan sulphate proteoglycans (HSPGs) [54]. Hondermarck and coworkers suggested this as a way to inhibit SARS-Cov-2 infection [92]. Binding to HSPGs at the cell surface was demonstrated to be the mechanism by which Lf was effective against HSV [64]. In this study Lf was only found to inhibit viral infection outside of the cell, whereas its digested peptide lactoferricin was transported intracellularly [47]. These studies demonstrated the significant role of the HSPGs in viral adhesion along with the role of Lf in its mitigation. The positive charge of Lf likely drives interaction with sulphate glycosaminoglycans. In sulfate inhibitor-treated Vero cells subsequently exposed to either arbovirus or Lf, a decrease in both viral plaque formation and Lf binding were observed [53]. Because the receptor for this virus is unknown, the authors speculate that Lf binding to plasma membrane surface glycosaminoglycans inhibit viral adhesion. The c-lobe region has also been demonstrated to be involved with viral receptor binding and inhibition. In a study on Hep C, lactoferricin, an N-lobe peptide, was not found to be effective against viral binding [62]. This has also been demonstrated against influenza viruses, including H1N1 and H3N2, in which the C-lobe and not N-lobe were responsible for virus binding of fusion proteins responsible for viral hemagglutination [52,56]. This group has also further investigated specific c-lobe peptides for potential anti-influenza therapeutics [93].

Another proposed mechanism of action for Lf against viruses is the inhibition of viral replication through induction of the immune response, specifically Th1 stimulation, interferon (IFN)-alpha/beta induction, B cell and natural killer (NK) cell activation [41,94]. The positive charge of Lf can easily interact and bind in a non-specific manner to immune cells, thereby leading to cell signaling activation including activation, differentiation and proliferation [95]. Activation of the IFN-β transcripts following Lf incubation, not direct viral binding, was recently found to be the mechanism behind anti-norovirus activity [96]. N-glycans isolated from bovine lactoferrin (bLf) has been demonstrated to lower TLR-8 activation, through interaction with the N-glycans on its dimer, the inhibitory effect was demonstrated to be as effective as chloroquine, a commonly prescribed anti-malaria and autoimmune drug [97]. In view of these anti-viral activities through immune modulation, Lf has also been investigated for use as a vaccine adjuvant. Adjuvants are bioactive substances added to, or alongside vaccines to enhance the immunogenicity, thus improving the performance of the vaccine. In neonatal mice, bLF administered as an adjuvant via intraperitoneal injection alongside influenza H1N1 vaccination was as effective in inducing an antibody response as the control (aluminum hydroxide) [98]. More research is needed to determine whether Lf consumed orally may have similar effects to improve vaccination response. Nonetheless, these immune modulatory activities of Lf have been demonstrated to target a variety of infectious diseases and inflammatory disorders.

#### 2.1.3. Infant Clinical Trials

Lf has been studied as an ingredient for the very low birth weight infant with early evidence suggesting some protection against NEC and sepsis [99,100]. A more recent randomized controlled trial (RCT) in the same population did not demonstrate these benefits [101,102]. It is possible that a lack of significant effects on Lf in those infants was related to substantial levels of Lf received by the control group, which was fed HM. Nonetheless, a Cochrane review found low-certainty evidence that Lf supplementation decreases late-onset sepsis in preterm infants [103]. However, with a large degree of heterogenicity between the studies, further investigation for its use in this population is warranted.

The addition of Lf to infant formula has demonstrated beneficial immune effects in healthy term infants. In an RCT of Lf added to infant (850 mg/L), infants exhibited significantly fewer lower RTIs [104]. In another infant formula trial, a lower prevalence of Giardia species, a pathogenic parasite, was observed. However, no decrease in the prevalence of diarrhea, the primary objective was demonstrated [105]. In another trial, in which Lf was added in combination with milk fat globule membrane (MFGM), both a 13% reduction in upper RTIs and a 25% reduction in cough was observed through 18 months of age [35]. In a study of stage 3, growing-up milk, children aged 12–32 months of age provided formula with added lactoferrin (48 mg/day) were demonstrated to have a lower prevalence of acute GI symptoms as well as having a lower number of sick days with acute respiratory symptoms [106].

A larger body of evidence exists for older children, in which both healthy and immunocompromised participants have been studied. However, not all studies are rigorously controlled, thus many of the outcomes need to be interpreted with caution. Additionally, both beneficial and null outcomes have been observed. For example, in a study in which children with recurrent RTIs were enrolled, a daily supplement with Lf (2.7 g) and curcumin (0.3 g) was found to improve immunity markers and reduce the number of RTIs. This trial did not have a control group and thus improvements were only seen from the enrollment baseline [107]. In a twelve-week study, dietary Lf (tablet or in yogurt, 100 mg per day) reduced the severity of rotaviral gastroenteritis [108]. In children between the ages of two and six who received Lf (70 mg/day) over 1 year in a day care setting, no differences in the prevention of enterovirus or rotavirus infection or serum IFN-gamma (IFN-γ) and interleukin-10 (IL-10) were observed [109]. Compared to consistent preclinical dose-dependent effects of Lf anti-viral activities [54], 70 mg/day may have been too low to observe an effect in children. In children receiving 100 mg/day in a day care setting, absences due to vomiting were reduced [110]. In HIV infected children, Lf has been studied as a potential intervention alongside anti-retroviral (ARV) treatment [111]. In these trials, alterations in plasma viral load as well as changes in the immune cell populations were observed. However, no control (without Lf) groups were included, making it challenging to determine the additive effect of Lf with ARV therapy.

#### 2.1.4. Clinical Trials in Adults

For adults, Lf has been studied in clinical trials designed to help with hepatitis C [112,113,114]. As oral direct anti-viral agents are now utilized as an effective treatment strategy, these studies will not be described in detail [115].

In a few small studies (with low sample size and a short duration), Lf supplementation has a demonstrated effect on immune cell population number/function as well as clinical immune system related outcomes. These outcomes demonstrate the secondary mechanism of action discussed above: inhibition of viral infections through modulating the immune response. Supplementation of Lf for four weeks modified either the number of polymorphonuclear leukocytes or CD T cell lymphocytes in 7 out of 10 healthy volunteers [116]. Healthy males (*n* = 8) enrolled in a three-week intra-individual repeated measures supplementation trial (7 days placebo, followed by 100 mg for 7 days, then 200 mg for final week) demonstrated a statistically significant increase in CD4+ and CD8+ cells as well as increased antioxidant capacity [117]. Lf supplementation (1 g/day for three weeks) alongside galacto-oligosacharides (GOS) and vitamin D were demonstrated to modulate pro-inflammatory cytokine production and plasmacytoid dendritic cells following a double blind RCT in elderly women [118]. Finally, a pilot study investigating an encapsulated lactoferrin in healthy adult males (*n* = 12) found decreased activation of CD4+ cells (measured by lowered CD69+ expression) during supplementation [119]. While these studies do not directly demonstrate measured clinical outcomes, modulation of these immune markers suggest that supplementation may aid individuals to support resistance to infectious diseases through these “activated’ cells.

The following studies report clinical outcomes in healthy adults due to Lf supplementation. In a double blind RCT of 90 adults with self-reported respiratory tract symptoms and infections, provision of a supplement with bovine Lf and IgG decreased the self-reported incidence of colds and the cumulative number of cold-related symptoms compared to the placebo group. The dose of bLF provided was 200 mg/day and 100 mg/day for the IgG provided over a period of three months (Vietta et al., 2013). While this study demonstrates some clinical benefit for Lf supplementation, the effect of Lf alone cannot be delineated as IgG was also included in the intervention. Finally, summer colds were found to be shortened due to Lf supplementation (200 and 600 mg) in healthy Japanese women [120].

Based on the literature examined, Lf appears to be a viable ingredient for consideration in reducing the risk of infection and in supporting an effective innate immune response. The studies demonstrate that dose, timing, and viral target are all important mediators for demonstrating efficacy. Additionally, in vitro evidence has demonstrated that heat treatment for food consumption processing can also reduce efficacy by rates between 3.8 and 87% [121]. Thus, care and attention to source, dose and processing is needed when determining effective nutraceutical uses for this supplement.

#### 2.1.5. Lf for SARS-CoV2

Discussion of Lf to strengthen resistance against SARS-CoV-2 is now underway [122,123,124]. A preliminary trial investigating a liposomal Lf supplement in COVID-19 patients examined symptoms pre- and post- study. While it appears symptoms improved following supplementation, statistical analysis was not performed. In addition, there was no control group. These limitations make any determination of efficacy challenging. The paper by Figueroa-Lozano et al., 2020, described earlier for Lf’s inhibition of TLR8 activation, also demonstrated similar efficacy to chloroquine, a drug that has been investigated for use in COVID-19 patients [125], although use of chloroquine for COVID-19 has not demonstrated clinical efficacy [126]. In vitro evidence is emerging for the use of Lf for SARS-CoV-2. In this trial, Lf inhibited Hu7 cell infectivity one hour and 24 h post infection. The same group also demonstrated that Lf potentiates the anti-viral activity of remdesivir and hydroxychloroquine (two drugs currently being investigated in COVID-19 patients) and suggests it may be an effective ingredient for combination therapy [127].

### 2.2. Milk Fat Globule Membrane (MFGM)

MFGM is the component of HM that delivers fat within a homogenous solution. It contains a variety of glycosylated proteins and lipids, which originate from the mammary epithelial membrane. MFGM has been studied both pre-clinically and clinically for its ability to support the immune system and aid resistance to infection [128]. The evidence to support the use of MFGM in infant nutrition has recently been reviewed [129,130].

#### 2.2.1. Mechanism of Action and Preclinical Evidence

There is some evidence that demonstrates the ability of MFGM to inhibit virus’, particularly rotavirus. The concentration of lactadherin, a protein embedded in the MFGM, in HM was negatively correlated with rotavirus infection. In this study, 200 mother–infant pairs in Mexico City were studied and the concentration of multiple proteins (mucin, lactadherin and butyrophilin) were measured and correlated to asymptomatic and symptomatic rotaviral infection, a significant protective effect of lactadherin was demonstrated for infants with asymptomatic infections. Mucin and butyrophilin concentrations were not found to be related [131]. Since then, mixed results for lactadherin and rotaviral vaccine seroconversion have been reported; one study demonstrated an association and another did not [132,133]. Bovine derived lactadherin fractions have also been examined, with a more limited efficacy than the HM isolated counterpart [134].

Whereas an older report demonstrated no correlation with rotavirus infection [131], new in vitro evidence suggests mucin proteins provide protection against viral diseases, including HIV and rotavirus [135,136]. The mechanisms by which inhibition is provided is likely through glycosylation of sialic acid residues [137,138], which has been demonstrated both in vitro and in vivo to inhibit rotavirus binding [139]. Within HM, the oligosaccharides are a large source of sialic acid residues, additional insights into the mechanisms behind sialylated-human milk oligosaccharides (HMO) against viral infections will be discussed in a following section.

MFGM isolates have also been demonstrated to strengthen the immune system against rotaviral infection. In a study investigating both buttermilk and whey cream derived MFGM, a fluorescent focus assay to measure rotaviral infectivity was performed. While all fractions of MFGM were demonstrated to have anti-rotaviral properties in a dose dependent manner, the cream derived isolate was slightly more effective [140]. This ingredient likely has a more diverse lipid fraction which may interact synergistically with the associated viral proteins. The significance of the lipid fraction in MFGM for inhibiting rotaviral infection was further demonstrated in an in vitro study in which bovine and ovine MFGM sources were examined. Within this study, a rotaviral-neutralizing activity was found for the cream-derived ingredients. However, both cream washing (diminished lipid content) and heat treatment (denatured proteins) lead to decreased effectiveness [141]. Thus, when considering these ingredients for use in nutrition products, the effect on processing of the bioactivity of the ingredient will likely come to bear.

#### 2.2.2. Clinical Evidence

Clinical evidence for MFGM on incidence of rotaviral infection is limited. Within one RCT in which a ganglioside enriched complex milk lipid was provided to older infants (8–24 months) for 12 weeks, a lower duration of rotavirus associated diarrhea and lower prevalence of illness was observed in the intervention group. Although a lowered incidence of rotaviral infection (the primary objective) was not demonstrated, authors stated the likelihood of the study being underpowered for a season of unseasonably low rotaviral infections [142]. Infants provided formula with added bovine-derived MFGM have demonstrated lower use of antipyretics [143] and rates of diarrhea, fever, or upper RTIs not different from their breastfed counterparts [144]. Whether these symptoms were viral or bacterial in nature cannot be determined. Nonetheless, they demonstrate the role this ingredient may have in modulating the immune system and potentially providing some support to resist viral infection.

### 2.3. Emerging Ingredients—Osteopontin and Glycerol Monolaurate

Osteopontin (OPN) is a phosphorylated glycoprotein present in a variety of tissues and bodily fluids, including HM. Its presence in milk has been ascribed to mediate cognitive, intestinal and immune development of neonates [145]. The protein has been extensively studied for its role in enhancing dendritic cell function and specifically TH17 cells. Endogenous OPN has been described for both its pathologic disease activities (in T-cell mediated tissue damage) and its protective effect on epithelial integrity [146]. Inhibition of rotaviral infection has been demonstrated with OPN in vivo. Within a OPN knock-out (KO) mouse model, suckling KO mice were susceptible to rotaviral infection, demonstrating prolonged diarrhea and an altered cytokine immune response [147]. However, in another OPN KO model, OPN was determined to be dispensable for protection against influenza and vaccinia virus [148]. Nonetheless, with its combined immunomodulatory and anti-adhesive activities, OPN could be a relevant ingredient to further investigate for anti-viral support.

Glycerol Monolaurate (GML) is a fatty acid monoester that has been described for its broad anti-microbial and immunological properties. Along with Lf, it is a component of HM, which was demonstrated to inhibit rhinovirus and cytomegalovirus in vitro [149]. Interestingly, the anti-viral properties of GML appear to only extend to enveloped viruses, including HIV and SIV [150,151,152,153,154] specifically at mucosal surfaces [155] as well as coronavirus [150]. This inhibition is likely through limiting viral adhesion, as demonstrated in a study in which GML hindered co-receptor CXCR4 binding of HIV [154]. Recently, it has been re-examined as an ingredient in HM, identified at a consistent concentration of 3000 mg/uL from six donor HM samples [156]. Within this in vitro study, removal of GML from HM samples created a decreased inhibitory action against the bacterial pathogen Staphylococcus aureus MN8 and the HM samples were demonstrated to inhibit toxic shock syndrome toxin-1 associated IL-8 secretion [156]. Thus, as GML has broad antipathogenic activities and is already a GRAS approved ingredient, further investigation of this ingredient for specific anti-viral applications is warranted.

### 2.4. Human Milk Oligosaccharides

Human milk oligosaccharides (HMO) are the third most abundant solid component in milk after lactose and lipids. These complex sugars are at the crosstalk between bacteria and the immune system. HMO are natural prebiotics and act as metabolic substrates for specific commensal bacteria, including *Bifidobacterium longum* subsp. *infantis* [157]. HMO stimulate the immune response and maturation of the epithelial cells [158], as well as affect the infant’s immune system, modulate immune cell populations and cytokine secretions [159]. The most abundant HMO, 2′fucosylactose (2′FL) is a neutral trisaccharide composed of L-fucose, galactose, and glucose units [160]. Recent clinical study has shown that infants fed formulas with 2′FL and GOS had 29–83% lower concentrations of plasma inflammatory cytokines and TNF-α than infants fed the control formula with GOS only [161]. HMOs display a broad spectrum of anti-viral protection, with structures resembling various cell surface carbohydrates [162]. Below we summarize studies that have shown that HMO act as decoy receptors for several viruses, as well as vaccine adjuvants.

#### 2.4.1. In Vitro and Preclinical Evidence

There are several mechanisms pointing at anti-viral properties of HMO, which include: balancing the Th1/Th2 cytokine response, stimulation of epithelial cells maturation, enhancing the growth of commensal bacteria and reduction of viral adherence to target cells. In this review, we focus on the effect of HMO to norovirus, rotavirus and influenza virus.

Two sialylated HMO, 3′sialyllactose (3′SL) and 6′sialyllactose (6′SL), possess anti-inflammatory activities and also resemble the host receptors, which may inhibit virus binding (i.e., act as a decoy) [163]. Using monkey kidney epithelial cells, researchers tested 2′FL, 3′SL and 6′SL in addition to GOS for infectivity of human rotaviruses, non-enveloped double-stranded viruses [164]. All oligosaccharides substantially reduced infectivity of two rotavirus strains. However, the maximum reduction was observed with 2′FL added at the onset of infection, while a combination of 3′SL and 6′SL was associated with the maximum reduction added during infection. Interestingly, all tested oligosaccharides reduced infectivity through an effect on the virus and not the tissue culture. The maximum reduction in infectivity observed with 3′SL and 6′SL is likely due to similarity of those sugars to the carbohydrate units of glycoconjugates on cell surfaces of mammalian epithelial cells. Azagra-Boronat and colleagues studied the gut dysbiosis induced during the rotavirus-associated diarrhea in neonatal rats [165]. They discovered that 2′FL increased TLR-5 and TLR-7 expression in the gut. Increased expression of those receptors was associated with higher count of *Lactobacillus* spp. and *Bifidobacterium* spp. In a separate study focused on sialylated HMO (including 3′SL and 6′SL), intranasal inoculation of a sialic acid bound to a polymeric compound (small molecules bonded together in long, repeating chains) reduced disease symptoms and decreased mortality in influenza-infected mice [166].

HMO also impact the immune system indirectly, via modulation of the microbiome. A diet containing 4 g/L HMO, consisting of 2′FL, 6′SL, lacto-*N*-neotetraose (LNnT), 3′SL, and free sialic acid, reduced duration of diarrhea in response to rotavirus infection in pigs [167]. Ileal tissue from the pigs fed HMO contained greater IFN-γ produced by Th1 cells, and increased IL-10 compared to control animals [167]. Thus, modulation of the microbiome by HMO, accompanied by mucosal immune responses resulted in reduction in rotavirus-associated diarrhea. Hester and colleagues tested HMO for anti-rotavirus activity in an established in vitro model and an in-situ piglet model [168]. They used a mix of neutral (LNnT and 2′FL) and acidic HMO (3′SL and 6′SL). Their study shows that while acidic HMO inhibited rotavirus infectivity in vitro, both neutral and acidic HMO decreased virus replication during acute rotavirus infection in situ. The authors concluded that neutral HMO (e.g., LNnT) were able to inhibit rotavirus binding within the milieu of the ileum likely via production of anti-inflammatory mediators. Dietary HMO were investigated in another study where they were shown to be more effective in altering systemic and GI immune cells in pigs compared to other prebiotic oligosaccharides [169]. Specifically, HMO-fed pigs had significantly more peripheral blood mononuclear cells, memory T cells and NK cells than control animals.

Weichert and colleagues elucidated the mechanisms through which HMO might inhibit the noroviruses, which are the dominant cause of acute gastroenteritis and are highly contagious [162]. Those researchers showed that both 2′FL and 3′-fucosyllactose (3′FL) structurally mimic histo-blood group antigens (HBGA), which are important factors in norovirus infections. Those two HMO bind at the equivalent pockets on the norovirus capsid, acting as natural decoys preventing virus from binding to host cells. This mechanism was confirmed in a study with 2′FL, which showed that it might function as a blocking component against multiple norovirus genogroups [170]. The expression of HBGA is a genetic factor that defines susceptibility to norovirus infection; interestingly, HBGA are synthesized through the action of fucosyltransferase 2 (FUT2), similarly to 2′FL [170]. Williams and colleagues further researched the activity of FUT2, which is encoded by the FUT2 gene and determines the secretor status and HBGA expression [171]. They discovered that maternal secretor status affected oral rotavirus vaccine immunogenicity. Further, infants of nonsecretor mothers were more likely to seroconvert (produce antibodies) than infants of secretors [171].

Gunther and colleagues [172] demonstrated that influenza A virus can be inhibited by 6′SL and 3′SL through conjugation to polymeric compounds. The authors suggested that both 3′SL and 6′SL are potent anti-virals for influenza as they mainly target envelope protein hemagglutinin, thereby preventing influenza virus from binding. Both sialated and neutral HMO were tested for prevention of influenza virus and RSV infections in vitro [173]. In the study, 2′FL decreased RSV viral load and cytokines associated with disease severity and inflammation in airway epithelial cells. Further, LNnT and 6′SL decreased influenza viral load in airway epithelial cells and 6′SL dose-dependently down-regulated TNF-α in RSV infected peripheral blood mononuclear cells.

#### 2.4.2. HMO Improve Vaccination Response

Several studies suggested that HMO have a role in improving vaccination response, acting as vaccine adjuvants. Using a mouse model, Xiao and coworkers showed that 2′FL improves both humoral and cellular immune responses to influenza vaccination; it also increased serum levels of vaccine-specific immunoglobulins IgG1 and IgG2a in a dose-dependent fashion [174]. In the same study, vaccine-specific CD4+ and CD8+ T-cells, as well as IFN-γ, were significantly increased in spleen cells in 2′FL-treated animals. In another study, researchers discovered that prebiotic mix consisting of 2′FL, short-chain galactooligosaccharides (scGOS) and long-chain fructooligosaccharides (lcFOS), improved influenza-vaccine-specific T-helper cell responses and B-cell activation. They correlated those observations with significant changes in the microbiome and its metabolites [175]. It is worth mentioning that microbiome composition stimulates production of virus-specific CD4+ and CD8+ T cells and influenza virus-specific antibodies [176]. The same combination of 2′FL, scGOS and lcFOS also the improved influenza vaccine-specific antibody response and modulated gut microbiota [177]. Interestingly, the antibody response was observed only in male mice.

Although the clinical evidence on the anti-viral effects of HMO are still limited, the published literature in preclinical models clearly points toward improving the immune response with these complex sugars. Considering HMO are undigestible, it is possible that any immunologic changes observed with HMO oral supplementation are correlated with microbial community structure and metabolites.

### 2.5. Omega 3 and Omega 6—Long Chain Polyunsaturated Fatty Acids

The fatty acid (FA) content of the immune cell membranes is modulated by the intake and type of dietary fats, in addition to genetics, and age of the individuals. Omega-6 (n-6) and omega-3 (n-3) are the two major families of polyunsaturated fatty acids (PUFAs). Specific types, such as linoleic acid (n-6) and alpha-linolenic acid (n-3) are described as essential and cannot be synthesized by animals. Once in the body, linoleic and alpha linolenic acids can be converted into other n-6 and n-3 fatty PUFAs, respectively. This conversion involves a series of desaturation and elongation reactions to yield to LCPUFA arachidonic acid (n-6) and eicosapentaenoic acid (EPA) and docosahexaenoic (DHA). In general, n-3 has immunosuppressive and anti-inflammatory effects, and n-6 plays a dual role with inflammatory and anti-inflammatory properties. Therefore, it is considered that their use can be beneficial in both inflammatory and autoimmune related diseases. The fatty acid type and n-6: n-3 ratio ingested through diet are also crucial in influencing host immune activity [178,179,180].

Foods typically high in n-3 fatty acids include fatty fish, algae, flax seeds, chia seeds, and walnuts, while n-6 fatty acids are typically found in high proportion in vegetable oils and seeds. It is important to note that HM is rich in long-chain polyunsaturated fatty acids (LCPUFAs) and that it is also affected by the mother’s dietary intake [179,181].

#### 2.5.1. Mechanisms of Action

Among the different mechanisms identified to explain the impact of LCPUFAs in the immune function, the synthesis of pro-resolving mediators is what can directly impact the pathogenesis of viral disease.

EPA and DHA are metabolized to metabolites known as specialized pro-resolving mediators (SPMs), which are known to directly modulate inflammation. While eicosanoids are known to be pro-inflammatory, resolvins and other SPMs counter pro-inflammatory cytokine production and can activate the anti-inflammatory process [182]. This activity is further stimulated through macrophage-mediated clearance of debris and attenuating neutrophil infiltration. Also, they help to attenuate pathological thrombosis and promote clot removal, [180,183] mechanisms emerging as a critical pathology of COVID-19 infection. While most COVID-19 clinical trials focus on “anti-viral” strategies, stimulating inflammation resolution may also be considered as a potential solution [31,184].

#### 2.5.2. Preclinical and Ex Vivo Evidence

Preclinical evidence suggests that dietary provision of LCPUFAs can modulate the immune response to reduce inflammation and viral infection. Within a mouse model, a specific DHA-derived protectin D1 isomer (PD1; 10S, 17S-dihydroxydocosahexaenoic acid) was found to markedly attenuate influenza virus replication via interference with the virus RNA nuclear export machinery. Within this study, PD1 was identified in self-limited resolving inflammatory exudates in vivo where it was demonstrated to regulate the innate local response and stimulate resolution of inflammation. PD1 inhibits virus replication, improves severe influenza infections and reduces influenza mortality [184]; as such it could be a novel target for severe influenza virus replication. Macrophage-derived extracellular vesicles (EVs) mediate long-lasting inhibitory effects on HCV replication, which may bridge the time until efficient adaptive immune responses are established and attenuated by PUFAs. In this study, exposure of macrophages to PUFAs, which are essential regulators of immune responses, dampened EV-mediated anti-viral immune responses. The anti-viral effect of EV’s from Caucasian and Japanese patients differed, which may be explained by different nutritional uptake of PUFAs [185]. Additional RCTs have further demonstrated the immune modulatory activities [186,187,188,189]. Thus, research demonstrates that PUFAs can provide targeted benefits against viral infections through direct modulation of the immune system.

#### 2.5.3. Clinical Trials in Infants

Consumption of LCPUFAs during the first thousand days of life is associated with altered inflammatory clinical outcomes [190]. Higher concentrations of LCPUFAs in HM are associated with reduced incidence of atopic diseases [191]. Maternal supplementation with LCPUFAs during lactation is associated with a reduction in the incidence of bronchopulmonary dysplasia and allergic rhinitis in preterm infants. [192].

Addition of DHA and ARA to infant formula is based on its presence in HM and is further deemed important due to these lipids being considered conditionally essential in infancy. Clinical trials have demonstrated both cognitive and immune improvements [193] with lowered incidence of RCTs in babies provided a formula enriched with DHA/ARA found in multiple studies [181,194,195,196].

These studies add to the increasing evidence of the potential contribution of DHA and ARA to improved respiratory health during infancy and childhood.

#### 2.5.4. Clinical Trials in Adults

An adequate intake of DHA and ARA supports the resolution of inflammation via the production of SPMs. In a healthy population, an intake of 250 mg EPA + DHA per day is recommended across multiple global, regional, and national experts [183].

Among countries published recommendations, the Federación Panamerica e lbérica de Medicina Critica y Terapia Intensiva stated that DHA and EPA quantity could be higher (2–3 g/day) in critically ill patients on mechanical ventilation with COVID-19 acute respiratory distress syndrome (ARDS). Moreover, larger amounts (4–6 g/day), only achievable with supplementary intake, have much more potent effects on cytokine secretion and inflammatory response [197].

Parenteral fish-oil emulsions containing substantial amounts of EPA and DHA have an excellent safety record in both critically ill adults and children, making them an appropriate candidate for off-label usage in clinical trials that investigate their usage in patients with COVID-19 [189]. Multiple reviews have explored the use of PUFA supplementation for COVID-19 patients and thus it is expected that research in this area will continue to develop [198,199]. Additionally, The European Society for Clinical Nutrition and Metabolism (ESPEN) recommendations for COVID-19 care includes the addition of omega-3 polyunsaturated fatty acids to improve oxygenation, but that the evidence is not yet considered strong [200].

In general, it is important to note that dosage, trial design, genetics, administration vehicle, lifestyle factors, lack of standardized formats and dosage (i.e., food versus supplement) and frequent lack of pre-study serum fatty acid level assessments, significantly limit the ability to compare outcomes across studies and to provide clear recommendations at this time. However, in a review by Dushianthan et al., evaluating ten RCTs in adults (aged 18 years or older) with acute respiratory distress syndrome, concluded that administration of Ω-3 fatty acids usually in combination with other bioactive nutrients led to reductions in the duration of mechanical ventilation and intensive care unit length of stay, along with improved oxygenation [188].

## 3. Ingredients Designed to Modulate the Microbiome

Maintaining a healthy immune system allows the body’s defense system to fight viruses. The microbiome plays a critical role in immune homeostasis as well as nutrient utilization in the host [201,202]. In contrast to an intact, balanced microbiota (eubiosis), a destabilized microbial community is referred to as dysbiosis [203]. Nutrients provided by HM are imperative for defining a healthy microbiota by supporting growth of beneficial bacterial strains [204]. In addition, the modification of the gut microbiota composition influences vaccine responsiveness [205]. Diet is one of the major environmental factors that affect both immune development and gut microbiota composition and function [205].

### 3.1. Probiotics

Probiotics are live microorganisms that, when administered in adequate amounts, confer a health benefit on the host [206]. Not all probiotics are equally effective and most benefits of probiotics are strain-specific [207]. Probiotics support immune function by adjusting the microbial balance and by direct interaction with the host immune system [208,209,210]. The gut microbiome has a critical impact on both systemic and mucosal immune responses, including the lungs [211]. Probiotics utilized in adults or children have been demonstrated to be safe and clinically effective in reducing the duration and severity of upper RTIs. Multiple clinical trials targeting the potential microbiome dysbiosis with COVID-19 are ongoing and the symptoms like diarrhea, nausea, vomiting and abdominal discomfort suggest that new SARS-CoV-2 has an impact on the gut microbiome [212]. Even though administered orally, probiotic strains can reduce the incidence and severity of viral RTIs. For example, probiotics modify the balance between pro-inflammatory and immunoregulatory cytokines that allow viral clearance while minimizing immune response-mediated damage to the lungs [213]. Many experimental studies in vitro and in animals show that specific strains of probiotics can provide immune support against viral infections. Select strains of *Lactobacillus paracasei* (*Lacticaseibacillus paracasei* according to recent reclassification [214]), *L. rhamnosus* and *L. plantarum,* were able to inactivate the vesicular stomatitis virus by directly binding the viral envelope with the infectivity of the virus diminished up to 68% in bacterial supernatants [215]. Different strains of lactic acid-producing bacteria reduced the titers of viruses including Ebola and cytomegalovirus [216]. The activity of probiotic strain *B. subtilis* against the influenza virus in vitro and in animals has also been investigated [217]. In this section, we focus on anti-viral activity of probiotic strains against respiratory viruses, including influenza and respiratory syncytial virus. We particularly emphasize the anti-viral activity of probiotics associated with pediatric nutrition or a healthy microbiome of breastfed infants.

#### 3.1.1. Mechanisms of Action of Probiotics

Probiotics may mediate anti-viral effects by inducing systemic immune responses via gut or by enhancing cellular immunity in the airways with increased activity of NK cells and macrophages [218,219,220]. Probiotic strains were shown to improve levels of type I IFN, increase the number and activity of antigen presenting cells, NK cells, T cells, as well as the levels of systemic and mucosal specific antibodies in the lungs [221]. In the gut epithelial cells, probiotics are recognized by TLRs [222] leading to an increase in the activity of immune cells such as leukocytes and neutrophils [223]. Toll-like receptors on probiotics include immunostimulatory substances such as lipoteichoic acid, peptidoglycan, and nucleic acid [224]. In summary, the mechanisms of action for probiotics related to anti-viral activity have previously been reviewed and include: (1) blocking binding and internalization of the virus; (2) production of metabolites and substances with a direct anti-viral effect; and (3) crosstalk with the host cells to establish the anti-viral protection [219]. These mechanisms enable probiotics to be effective both directly in reducing viral adhesion and indirectly for GI symptoms of viral infections. Probiotics might also help reduce the risk of secondary infections due to microbial translocation in severe COVID-19 cases [225].

#### 3.1.2. In Vitro and Preclinical Evidence

Researchers studying mechanisms of action of probiotics have focused on direct binding to the virus and inhibition of the virus attachment to the host cell receptor. In a recent in vitro study, probiotics were shown to block the adherence of rotavirus to monkey kidney cells [226]. Those researchers tested several strains of *Lactobacillus* spp. and *Bifidobacterium* spp., two major bacterial genera linked to health benefits, in tissue culture before infecting them with rotavirus. Instead of interaction with cellular receptors and blocking the attachment of the virus to the cell surface, their results show that anti-viral activity observed with probiotics occurs directly with the viral particle. Vlasova et al. used a neonatal piglet model to study the effects of colonization with *Lactobacillus rhamnosus* GG (LGG; *Lacticaseibacillus rhamnosus* GG according to recent reclassification [214]) and *B. animalis* subsp. *lactis* (BB12) on 3-dose vaccination with attenuated rotavirus. In that study, probiotics together with vaccination completely protected the animals from rotavirus-associated diarrhea [227]. The authors concluded that a combination of LGG and BB12 exert anti-inflammatory and anti-viral actions via TLR signaling.

Influenza virus is a major causative agent of both upper and lower RTIs [16,17]. Multiple probiotics have been studied for its effectiveness against influenza. *L. rhamnosus* GG (LGG), a probiotic used in pediatric nutrition, applied intranasally was shown to help against influenza infection in mice by stimulating innate immune responses directly in the respiratory epithelium [228]. *Bifidobacterium bifidum*, bacterial species associated with healthy, breastfed infants, has shown positive effects against influenza A H1N1 virus, inducing humoral and cellular immunity, which included lower production of IL-6 as well as higher survival rate in mice [229].

#### 3.1.3. Clinical Evidence

Studies have shown that probiotics modulate the innate and adaptive immune responses, resulting in increased levels of serum IgG and secretory IgA targeting enteric pathogens. Sindhu and colleagues have demonstrated that LGG supplementation decreased the episodes of rotavirus diarrhea [230]. They tested the effect of LGG in 6-month to 5-year-old children with rotavirus infection. Indeed, LGG had an immunomodulatory effect, which included an increase in circulating IgG levels. Also, fewer children had rotavirus-associated diarrhea after LGG intervention. Those mechanisms were extrapolated in a preclinical study, where LGG enhanced intestinal permeability and stimulated mucin expression [231]. BB12 was used in another study focused on antibody response where anti-poliovirus-specific IgA and anti-rotavirus-specific IgA were assessed in 6-week-old healthy, full-term infants [232]. BB12 significantly increased anti-poliovirus-specific IgA and showed the tendency of increased anti-rotavirus-specific IgA after 6 weeks of intervention. Probiotics were also shown to be effective anti-virals in preterm infants. An RCT including 94 preterm infants showed that LGG given immediately after birth lowered the incidence of virus-associated RTIs by 2- to 3-fold compared to placebo [233]. LGG was associated with the reduction of rhinovirus-associated episodes in that study.

Clinical studies with probiotics have reported a modest effect on the antibody response to vaccination in adults. Trials in older subjects are largely inconsistent and data are limited [234]. It has also been demonstrated that ecological fitness, antipathogenic effects in-vitro, and immunomodulatory effects are strongly influenced by the age of the host [235]. These data open the possibility of altering the gut microbiota with symbiotic prebiotics and probiotics might offer novel and cost-effective methodologies to reduce the risk of viral infections.

#### 3.1.4. Probiotics as Vaccine Adjuvants

Considering immunomodulatory effects of probiotics, several studies focused on studying the impact of probiotic supplementation on antibody responses and other outcomes following vaccination. For example, LGG was effective in supporting the immune response against the H3N2 strain in an influenza virus vaccine trial [236]. Moreover, ingestion of *L. fermentum* CECT5716, a strain used in infant nutrition, resulted in lower influenza-like illness in adults, increased proportion of NK cells in blood, significantly higher TNF-α, and increased anti-influenza-specific IgA and IgM after influenza vaccination [237]. The consumption of BB12 also showed significantly greater increase in influenza virus vaccine-specific IgG antibodies in plasma and secretory IgA in saliva [238]. Bianchini and colleagues [239] conducted an interesting study, which included a 3-month intervention with LGG in children and adolescents with type 1 diabetes. The LGG supplementation lead to an increased immune response to influenza vaccine through reduction in inflammatory responses. While the administration of LGG did not improve the humoral responses to an influenza vaccine, the probiotic had an anti-inflammatory effect. Based on current state of knowledge, probiotic supplementation may hold a great promise for improving influenza vaccine efficacy.

### 3.2. Postbiotics

Postbiotics are an actively emerging functional food within the microbiome modulation category. While there has been no official definition of postbiotics, they have been tentatively defined as the following: any factor resulting from the metabolic activity of a probiotic or any released molecule capable of conferring beneficial effects to the host in a direct or indirect way. Postbiotics can be thought of as cutting out the “middle man”, they provide the biologically active component, potentially removing the need to have colonization (probiotics) or stimulate the growth of commensal bacteria (prebiotics) [240,241]. Additionally, they help to bypass live probiotic safety concerns with infants and antibiotic resistance [242,243]. Postbiotics can be microbial components in the form of non-viable cells (heat-killed bacteria, UV-inactivated cells) or cell components (DNA, RNA, teichocic acid, polysaccharides) [241]. They could also be compounds derived from microbial action, either synthesized metabolites (short chain fatty acids, vitamins, peptides, bacteriocins) or produced by enzymatic action (peptides from milk proteins) [241]. Roggero and colleagues [244] suggested that many beneficial activities associated with breast milk may be provided by postbiotics, including metabolites from lactic acid bacteria.

#### 3.2.1. Mechanism of Action

Postbiotics have been investigated for use in pediatrics for the suppression of infectious disease by directly interacting with the mucosal innate immune system (through TLR’s and NOD signaling pathways) as well as through bactericidal activities [245]. The impact heat killed *L*. *paracasei* CBA L74 (CBA L74) on the mucosal barrier using a standard Caco-2 human epithelial cell model was examined. Following 48 h of incubation with CBA L74 at varying concentrations there was stimulation of cell growth and differentiation, tight junction protein expression, mucin-2 expression and mucus layer thickness indicating improved mucosal barrier function [246]. These preclinical findings were further examined in similar models. An up-regulation of human beta defensin 2, cathelicidin, IL-37 was demonstrated in a dose-dependent fashion when CBA L74 was provided to a human enterocyte cell line [247]. Finally, in a mouse DSS- experimental colitis model the CBA L74 treated mice showed much higher survival and less colon injury than placebo treated animals [248].

Postbiotics have been demonstrated to have immune supportive activity against influenza, rotavirus, and human immunodeficiency virus. MDCK cells have been utilized to investigate cell-free supernatant of MRS fermented by lactic acid bacteria for both H1N1 (in which *Lactobacillus plantarum* YML009 was utilized) and the avian influenza (H9N2) (in which *Leuconostoc mensenteroides* YML003 was utilized). In both in vitro studies, anti-viral activity was found, with *L*. *plantarumarum* YML009 being more effective than Tamiflu in the H1N1 infection model [249,250]. Preclinical studies with fermented infant formula containing postbiotics derived from *B. breve* C50 and *Streptococcus thermophilus* 065 showed prolonged dendritic cell survival and maturation and induced high IL-10 production through TLR-2, suggesting immune regulatory functions associated with postbiotics. Postbiotics derived from these two strains improved the epithelial barrier function and stimulated Th1 response in mouse models suggesting the involvement of postbiotics in host immune function [251,252]. The same postbiotics were shown to reduce the risk of rotavirus-associated diarrhea [253]. This dietary intervention reduced two of the clinical symptoms of diarrhea (incidence and severity) and improved the immune response against rotavirus by increasing anti-rotavirus IgG and intestinal anti-rotavirus IgA antibodies in the sera. In addition, the fermented milk with postbiotics was able to bind the virus and reduce its clearance. Aria and colleagues used heat-killed *L. paracasei* MCC1849 to study influenza infection. Those researchers showed that postbiotics increased the IgA production in the small intestine and serum, facilitating protection against influenza virus infection in mice [254]. Martin et al. evaluated heat-killed commensal breastmilk bacteria and their cell-free supernatants for capacity to constrain HIV-1 infection in vitro. Their findings showed that postbiotics, obtained mainly from *Lactobacillus* and *Pediococcus*, inhibited HIV-1 infection. This study suggests a possible role for these bacteria and their metabolites in mucosal protection against HIV-1 in the breastfeeding infant [255].

#### 3.2.2. Clinical Evidence

In a clinical trial, LGG (both heat-killed and viable) significantly improved diarrhea recovery in young children under four [256]. A recent systematic review has identified seven RCT’s with 1740 children under the age of four comparing use of postbiotics to a placebo or no intervention. From this set of studies multiple non-viable (heat killed) ingredients were examined, including *L. acidophilus* LB (four RCTs which demonstrated reduction in diarrhea), *L. paracasei* CBA L74 (2 RCTs) and a study with *B. breve* C50 and *Streptococcus thermophilus* [257].

To date, heat or UV killed probiotic cells and their metabolites have been primarily researched for human consumption. However, there is growing interest in the use of synthesized molecules; including short chain fatty acids and peptides for use as functional foods. Butyrate, a short chain fatty acid, has been shown to directly modulate T-cell immunity and may be a profound ingredient to help reduce the risk of autoimmune disorders [258,259]. Butyrate has been measured within HM at a level demonstrated to have preclinical effects on gut barrier function and response to food allergens. The location of Butyrate (present in HM or produced by the colon) likely influences where it is absorbed (small or large intestine) and further investigations into its various luminal roles are needed. Butyrate absorption and its potential effects on intestinal health has recently been reviewed [260]. While palatability is currently an issue, innovations in butyrate production could provide impactful nutrition solutions in the near future [261]. Casein hydrolyates also have emerging evidence in the area of direct immune modulation [262]. With continued need for effective nutritional strategies to support the immune system, it is anticipated that interest and research in these postbiotic ingredients will continue to add to the evidence base.

## 4. Conclusions

In this review we presented key studies focused on anti-viral properties of nutritional ingredients inspired by HM and infant nutrition. Disease severity of COVID-19 ranges from mild flu-like symptoms, to pneumonia, and potentially life-threatening complications and multiple organ failure. Although the transmission of SARS-CoV-2 occurs mainly via respiratory droplets, the gut may also contribute toward the pathogenesis of COVID-19 and nutrients within the gut may modulate the ability to resolve infection. Indeed, research suggests that nutrients inspired by HM and infant nutrition research can improve the immune response to viruses and/or prevent direct viral binding inhibition. However, further research is needed. Nonetheless, HM-inspired nutrition could play a role in strengthening the immune system to reduce the risk and aid management of viral diseases.

## Data Availability

Not applicable.

## Abbreviations

2′FL	2′fucosylactose
3′SL	3′sialyllactose
6′SL	6′sialyllactose
ACE2	Angiotensin-converting enzyme 2 receptor
CDC	Centers for Disease Control and Prevention
COVID-19	Coronavirus disease 2019
FOS	Fructooligosaccharides
FUT2	Fucosyltransferase 2
GML	Glycerol monolaurate
GOS	Galactooligosaccharides
GRAS	Generally Recognized as Safe
HM	Human milk
HSPG	Heparan sulfate proteoglycans
HSV	Herpes simplex virus
IFN	Interferon
IL-10	Interleukin-10
Lf	Lactoferrin
LNnT	Lacto-*N*-neotetraose
MAPK	Mitogen-activated protein kinase
MFGM	Milk fat globule membrane
PUFA	Polyunsaturated fatty acids
RCT	Randomized controlled trial
RSV	Respiratory syncytial virus
RTI	Respiratory tract infections
SARS-CoV-2	Severe acute respiratory syndrome coronavirus 2
TLR-2	Toll-like receptor-2
WHO	World Health Organization
